# Incisor irregularity and dental arch dimensions changes in subjects with different severity of anterior crowding: a 37-year follow-up

**DOI:** 10.1186/s40510-023-00461-8

**Published:** 2023-03-20

**Authors:** Caroline Martins Gambardela-Tkacz, Gabriela Alcaraz, Paula Cotrin, Karina Maria Salvatore de Freitas, Willana Moura, Guilherme Janson, Daniela Garib, Marcos Roberto de Freitas

**Affiliations:** 1 Department of Orthodontics, Bauru Dental School, University of São Paulo, Alameda Octávio Pinheiro Brisolla 9-75, Bauru, SP 17012-901 Brazil; 2 Department of Orthodontics, UNINGA University Center, Maringá, PR Brazil

**Keywords:** Malocclusion, Tooth extraction, Recurrence, Secondary prevention, Longitudinal studies

## Abstract

**Background:**

Occlusal stability is one of the goals of orthodontic treatment, and keeping teeth aligned in the long term is a challenge for the orthodontist. This study aimed to compare the long-term incisors irregularity and dental arches dimensions changes in subjects treated with 4 premolar extractions with different pretreatment Little's irregularity index (LII). The knowledge of long-term outcomes is evidence-based information for the prognosis of future treatments.

**Methods:**

In total, 41 treated subjects were divided into two groups according to mandibular Little irregularity value at pretreatment (mild or severe). The maxillary and mandibular LII, transversal, and longitudinal widths were assessed at pretreatment, posttreatment, and 37-year posttreatment. Chi-square and independent t tests were used for intergroup comparison.

**Results:**

The groups presented similar behavior for all stages of maxillary and mandibular arch dimensions changes. Maxillary irregularity was corrected in both groups after treatment, and the alignment was acceptable in the long term. In the mild group, the mandibular incisor irregularity returned to pretreatment values in the long term. The mandibular LII increased in the severe group but did not return to pretreatment values in the long term.

**Conclusions:**

The mild crowding group presented proportionally more relapse of mandibular incisor irregularity than the severe crowding group in the long term. Even so, the correction of mild and severe crowding with the extraction of 4 premolars showed satisfactory results in the long term, even with the presence of maturational changes and relapse.

## Introduction

The occlusal traits tend to relapse gradually over time [1–5]. About half of the total relapse takes place in the first two years after retention, remaining relatively stable from 5-year postretention [1]. The mandibular anterior irregularity shows fast and continuous increases, even exceeding the initial scores [1].

Anterior crowding relapse after orthodontic treatment is a typical orthodontic patient's chief complaint in private practice [6–8]. The etiology of relapse is multifactorial and still is not entirely understood. The relapse could be associated with several factors, as follows: unfavorable growth of the basal bones, tissue response after the release of orthodontic forces, tongue muscles imbalance, poor relation between lips and cheeks [9], changes in the shape of the dental arch, changes in the cortical bone thickness and mandibular alveolar bone structure, periodontal and occlusal factors, oral and soft tissue pressures, late growth and patients compliance with the retainers [10]. These changes may also result from expected age-related effects. The aging of the occlusion is a physiological phenomenon that promotes continuing dentoalveolar changes throughout adult life [11]. No specific characteristics, variables or kinds of treatment could be used to predict long-term results after orthodontic treatment [6–8].

Dental crowding is one of the most frequent malocclusions in the population, and it is a common orthodontic problem [12]. Moderate crowding is also observed among indigenous populations with marked tooth wear that have never received orthodontic treatment [13]. Accordingly, relapse of the anterior segment during the postretention periods is predictable and sometimes, these long-term changes can be associated with treatment failures [14]. The orthodontic literature states that anterior irregularity worsens over the years and the dental arch dimensions decrease, both in orthodontically treated and untreated cases [2, 3, 5, 15, 16].

Long-term studies on relapse of occlusal characteristics show that changes in dental relationships many years after treatment are common and must be taken into account when planning orthodontic treatments [2, 3, 17]. These changes occur both in patients treated with and without dental extractions [2, 3, 15, 17, 18]. Recent studies showed that extraction treatments have better long-term occlusal results than no extraction ones [2, 18]. It is well known that the long-term response to mandibular anterior alignment is unpredictable [6].

The life expectancy of the population is increasing. The challenge of the orthodontist is to conduct an orthodontic treatment with excellent results and proportionate oral health-related quality of life for the patient. The knowledge of long-term outcomes is evidence-based information for the prognosis of future treatments [8]. On our concern, it is essential to know whether the amount of initial irregularity influences long-term occlusal changes in patients treated with 4 premolar extraction. Most studies focus mainly on the relapse of lower anterior crowding with shorter long-term follow-up times [1, 4, 17, 19]. Based on these, this study aimed to compare long-term incisors irregularity and dental arches dimensions changes in subjects treated with 4 premolar extractions with different pretreatment Little's irregularity index more than 30-year postretention.

## Material and methods

This nonrandomized longitudinal retrospective cohort study was approved by the Ethics Research Committee of Bauru dental School (number 3.834.688), and written consents were obtained from all subjects.

The sample size calculation was based on an alpha significance level of 5% and a beta of 20% to achieve an 80% test power to detect a mean difference of 2 mm, with a standard deviation of 1.26 mm in mandibular irregularity index at follow-up [15]. Thus, the sample size calculation determined that at least 7 subjects were necessary per group.

The sample was selected according to the following inclusion criteria: Class I or any severity of Class II malocclusion at the beginning of the treatment, complete orthodontic treatment with fixed edgewise appliances; treatment protocol with 4 first premolars extractions or with maxillary first and mandibular second premolar extractions; the presence of full permanent dentition, no tooth agenesis or anomalies; removable maxillary appliance (Hawley plate) worn for at least 1-year posttreatment and mandibular fixed canine-to-canine retainers worn for at least 1–5 years after treatment; complete orthodontic records from pretreatment (T1) and posttreatment (T2). The exclusion criteria were a history of new orthodontic treatment. The eligible patients received a letter or a message on social media containing information about the research and an invitation to a further follow-up examination.

The sample comprised 41 treated subjects by graduate students in the 1970s and 1980s from the files of the Department of Orthodontics, Bauru Dental School, University of São Paulo, Bauru, São Paulo, Brazil. The sample was divided into 2 groups, group mild crowding (LII ≤ 6 mm) and group severe crowding (LII > 6 mm), according to the mandibular Little irregularity value at pretreatment (T1).

Orthodontic mechanics in both groups included fixed edgewise appliance 0.022 × 0.028-in slot with stainless steel archwires. Nickel–titanium archwires were not used in this sample. Extra-oral headgear was used as anchorage to maintain Class I and to correct the Class II molar relationship; anterior teeth were retracted with a rectangular archwire and elastic chains; Class II elastics were used when necessary.

Long-term follow-up examinations were performed by 2 orthodontic postgraduate students (CMGT and PPCS) from October 2017 to October 2019 at Bauru Dental School, University of São Paulo.

Dental casts were scanned and the images were acquired with the 3Shape R700 3D scanner (3Shape A/S, Copenhagen, Denmark). The images were saved in STL format, compatible with software for 3D images.

**Fig. 1:**
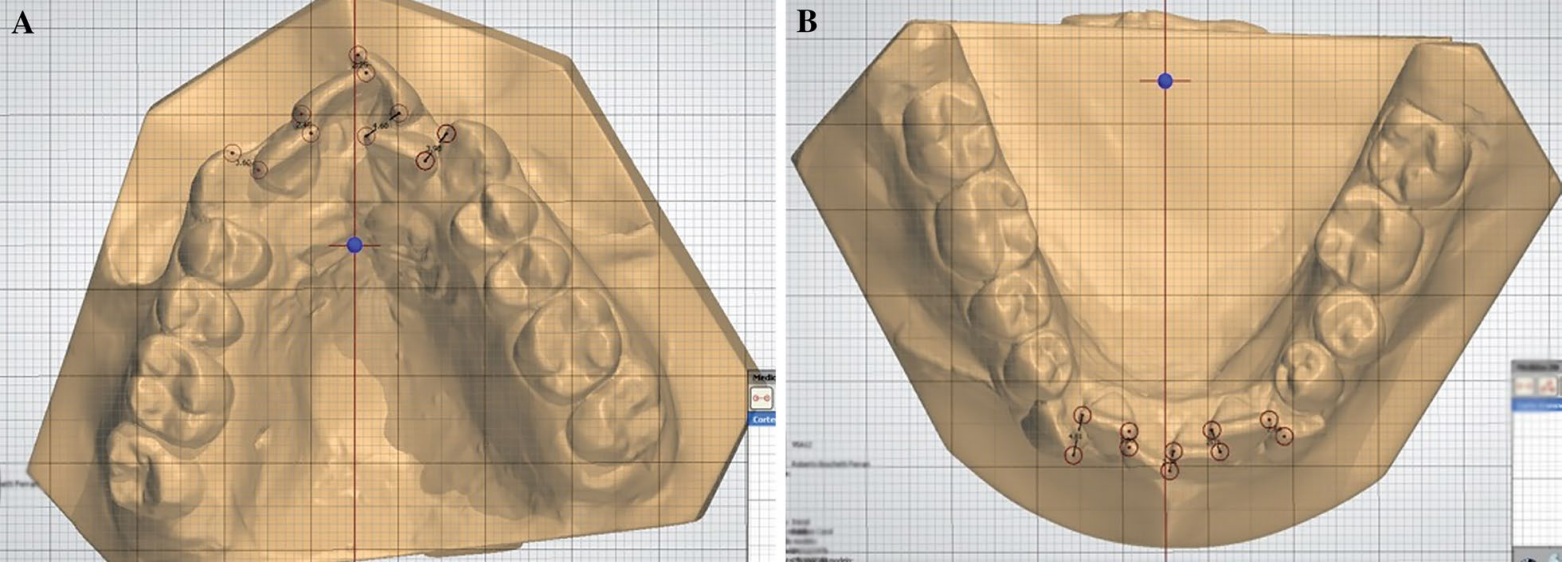
**A** Measurement of maxillary Little's irregularity index. **B** Measurement of mandibular Little's irregularity index

**Fig. 2:**
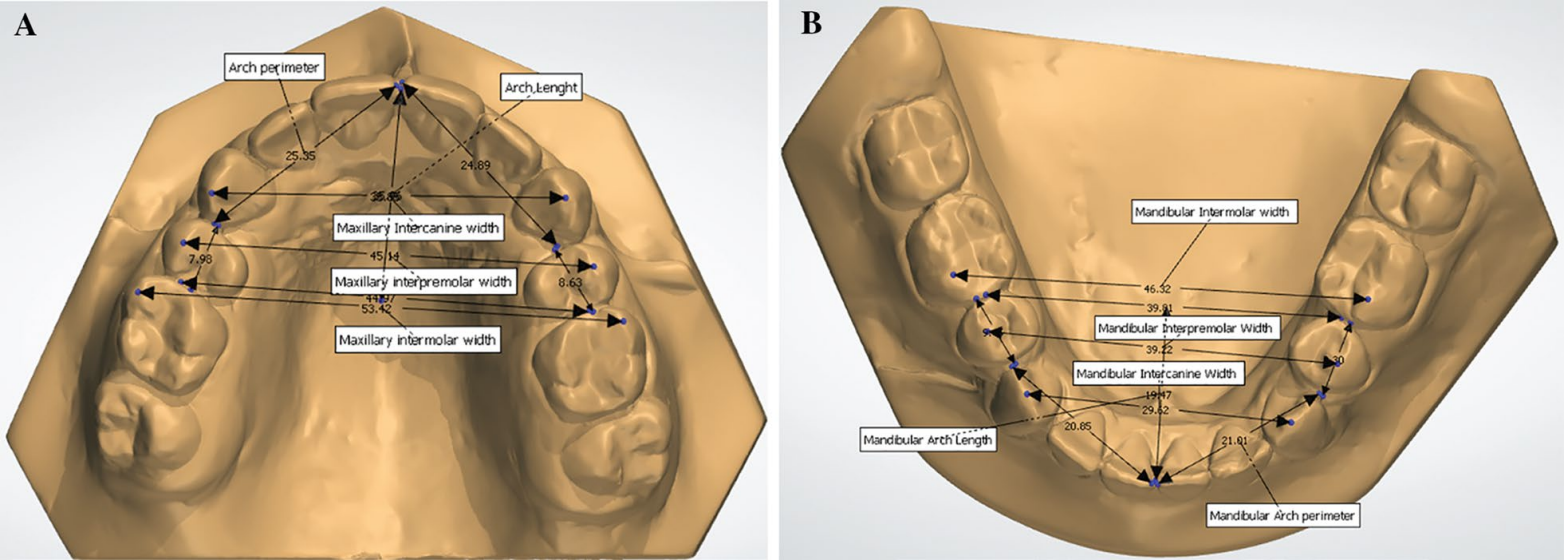
**A** Measurements of maxillary transversal and longitudinal widths. **B** Measurements of mandibular transversal and longitudinal widths

Measurements were taken in maxillary and mandibular arches and included: Little irregularity index (LII): the quantitative method of assessing anterior irregularity. The sum of the five linear displacements of the anatomic contact points of each incisor from the adjacent tooth's anatomic point represents the relative degree of anterior irregularity [20]. In this study, the Little irregularity index was modified to measure the irregularity of the maxillary anterior teeth. A virtual plane parallel to the occlusal plane was created to avoid the vertical difference in height between the displacements of the contact points. This plane was defined as passing through an idealized contact point between the incisors and the mesio-palatal cusp tips of the first molars.[21] (Fig. 1A, B) Intercanine width: distance between the cusp tips of the permanent canines; interpremolar width: distance between the buccal cusps tips of the first or second premolars; intermolar width: distance between the mesiobuccal cusp tips of the first molars; when the dental cusps tips presented signs of worn in posttreatment follow-up measures (T3), our reference point was the center of the worn cusp. Arch length: the distance from the lingual contact point between the central incisors to a line connecting the mesial contact points of the first molar from one side to the other; arch perimeter: the sum of the segments from mesial permanent first molar contact to the distal canine and from the distal canine contact to the mesial central incisor contact, on both sides (Fig. 2A, B). These variables were measured in millimeters. One operator (CMGT) performed all measures in Ortho Analyzer 3D (3Shape A/S, Copenhagen, Denmark).

The quantitative values in millimeters to calculate the orthodontic correction were obtained by reducing LII measures at post- and pretreatment (T2-T1). Relapse can be defined as a physiological tendency of teeth to move from the positions it was placed after treatment [22]. To measure the relapse, we deduct LII values at long-term follow-up from LII values at posttreatment (T3-T2) (Table 3).

### Error

To determine the error involved in the method and the reliability of the results, 30% of the sample was randomly selected and remeasured after 15 days. The random errors were calculated according to Dahlberg's formula [23]. The systematic errors were evaluated with paired t tests at *P* < 0.05.[24].

### Statistical analyses

The Kolmogorov–Smirnov test verified the normal distributions. The data showed normal distribution. Chi-square tests compared ages, treatment, follow-up and retention times, sex distribution, and initial malocclusion. Independent t tests were applied for intergroup comparison.

A multiple linear regression analysis was performed to evaluate the influence of sex, type of malocclusion, age at long-term posttreatment follow-up, retention time, and initial mandibular Little irregularity index in the mandibular anterior crowding relapse.

The statistical tests were performed with Statistica software (Statistica for Windows, version 12.0, StatSoft, Tulsa, Okla, USA), and the results were considered significant at *P* < 0.05.

## Results

The random errors ranged from 0.10 mm (maxillary interpremolar width at T2) to 1.13 mm (mandibular arch length at T2) and were within the acceptable ranges.[23] Maxillary arch length at T1, mandibular intercanine width at T1, maxillary intermolar width at T2 and maxillary interpremolar width at T3 showed statistically significant systematic errors.

The mandibular Little's irregularity index (LII) at pretreatment was the variable used to divide the sample into two groups as follows:

### Group mild crowding

There are 16 subjects (9 females and 7 males) with mandibular Little irregularity index less or equal than 6 mm at the beginning of the treatment (3.31 ± 2.10 mm). The mean ages at pretreatment, posttreatment and postretention stages were 13.45 ± 1.57, 15.98 ± 1.97 and 54.93 ± 3.78, respectively. The mean treatment, retention and long-term follow-up times were 2.53 ± 0.72, 1.80 ± 0.73 and 38.95 ± 2.88 years, respectively. Four patients presented Class I malocclusion (9.76%), and 12 (29.26%) had Class II malocclusion.

### Group severe crowding

There are 25 subjects (17 females and 8 males) with mandibular Little irregularity index greater than 6 mm at the beginning of the treatment (9.95 ± 2.65 mm). The mean ages at pretreatment, posttreatment and postretention stages were 13.25 ± 2.20, 15.40 ± 2.25 and 52.85 ± 6.23, respectively. The mean treatment, retention and long-term follow-up times were 2.15 ± 0.43, 2.48 ± 1.27 and 37.45 ± 5.23 years, respectively. Twenty (48.78%) patients presented Class I malocclusion, and 5 (12.20%) had Class II malocclusion.

The groups were comparable regarding ages, follow-up time evaluation, retention time and gender distribution. The mild crowding group (LII < 6 mm) showed a longer treatment time than the severe group, and the mild group presented more Class II malocclusion subjects. At pretreatment, the severe group presented more subjects with Class I malocclusion (Table 1).

**Table 1:** Results of intergroup comparability of the ages, treatment, follow-up, retention times, gender and initial malocclusion distribution (independent t test and Chi-square test)

Variables	Group 1 Mild crowding (*N* = 16)		Group 2 Severe crowding (*N* = 25)		*P*
	Mean	SD	Mean	SD	
Initial age (T1)	13.45	1.57	13.25	2.20	0.756
Final age (T2)	15.98	1.97	15.40	2.25	0.409
Follow-up age (T3)	54.93	3.78	52.85	6.23	0.239
Treatment time (T2-T1)	2.53	0.72	2.15	0.43	0.046*
Follow-up time (T3-T2)	38.95	2.88	37.45	5.23	0.302
Retention time	1.80	0.73	2.48	1.27	0.062
Gender distribution	9 (21.95%) female 7 (17.07%) male		17 (41,46%) female 8 (19.51%) male		0.447^Chi^
Initial malocclusion	12 (29.26%) Class II 4 (9.76%) Class I		5 (12.20%) Class II 20 (48.78%) Class I		0.000* ^**chi**^

^*^Statistically significant at *p* < 0.05

^chi^Chi-square

Both groups presented similar behavior for maxillary and mandibular arch dimensions for all stages. There were no differences for maxillary variables between groups at pretreatment, posttreatment and postretention. The mandibular Little's irregularity index (LII) was greater in the severe group at pretreatment (9.94 ± 2.65) than in the mild group (3.31 ± 2.10) (Table 2).

**Table 2:** Results of intergroup comparability at pretreatment (T1) posttreatment (T2) and postretention (T3) (independent t tests)

Variables (mm)	Group mild crowding (*N* = 16)	Group severe crowding (*N* = 25)	*P*	Group mild crowding (*N* = 16)	GROUP Severe Crowding (*N* = 25)	*P*	Group mild crowding (*N* = 16)	Group severe crowding (*N* = 25)	*P*
	Mean (SD)	Mean (SD)		Mean (SD)	Mean (SD)		Mean (SD)	Mean (SD)	
	T1			T2			T3		
*Maxillary dental casts measurements*									
Little's irregularity index	6.84 (2.97)	8.40 (4.10)	0.195	0.37 (0.68)	0.12 (0.41)	0.154	1.55 (1.95)	2.71 (2.38)	0.111
3–3 width	34.20 (2.50)	34.57 (2.25)	0.650	34.93 (1.76)	34.8 (1.86)	0.922	33.02 (4.02)	34.02 (2.10)	0.303
5–5 width	44.79 (2.66)	43.81 (2.18)	0.218	43.46 (1.64)	42.7 (1.97)	0.216	41.96 (2.41)	41.55 (2.31)	0.591
6–6 width	49.39 (2.62)	49.09 (2.10)	0.689	48.28 (1.43)	47.79 (2.45)	0.473	47.51 (2.40)	47.37 (2.76)	0.877
Arch length	27.89 (2.71)	28.08 (2.64)	0.821	22.71 (3.51)	22.0 (2.91)	0.537	20.11 (2.32)	21.18 (1.62)	0.089
Arch perimeter	70.66 (5.98)	72.16 (5.29)	0.404	61.91 (3.06)	62.9 (2.67)	0.254	58.86 (3.72)	60.47 (3.49)	0.170
*Mandibular dental casts measurements*									
Little's irregularity index	3.31 (2.10)	9.94 (2.65)	0.000*	0.58 (0.97)	0.48 (0.82)	0.720	3.67 (2.11)	4.60 (3.05)	0.292
3–3 width	26.53 (2.01)	25.73 (2.24)	0.253	26.7 (1.33)	27.15 (1.38)	0.345	24.23 (2.70)	25.36 (2.32)	0.160
5–5 width	39.43 (3.17)	37.17 (2.20)	0.011*	35.40 (1.92)	35.51 (1.73)	0.846	34.48 (2.48)	34.06 (2.04)	0.551
6–6 width	44.05 (2.01)	42.71 (2.37)	0.071	40.7 (1.43)	40.1 (2.53)	0.379	41.78 (2.91)	40.38 (2.89)	0.140
Arch length	23.11 (1.87)	22.72 (2.52)	0.595	17.87 (1.93)	18.2 (2.33)	0.608	15.58 (1.76)	16.93 (1.98)	0.032*
Arch perimeter	62.20 (5.53)	63.76 (3.11)	0.255	52.76 (3.03)	53.6 (2.27)	0.311	49.04 (3.11)	50.47 (3.35)	0.180

^*^Statistically significant at p < 0.05

In the mild group, the maxillary irregularity was corrected with treatment (from 6.84 ± 2.97 to 0.37 ± 0.68), and the alignment was acceptable in the long term (1.55 ± 1.95). The mandibular Little irregularity index was corrected with treatment (from 3.31 ± 2.10 to 0.58 ± 0.97) and relapsed to the baseline values in the long term (3.67 ± 2.11). The mild group presented mandibular interpremolar width (39.43 ± 3.17 mm) greater than the severe group (37.12 ± 0.011 mm) at pretreatment (T1). In the severe group, the maxillary irregularity was corrected with treatment (from 8.4 ± 4.10 to 0.12 ± 0.41 mm) and relapsed over the long term but did not return to baseline values (2.71 ± 2.38 mm). The mandibular LII was corrected with treatment (from 9.94 ± 2.65 to 0.48 ± 0.82) and relapsed over the long term but did not return to pretreatment values (4.60 ± 3.05). The severe group presented mandibular arch length (16.93 ± 1.98 mm) greater than the mild group (15.58 ± 1.76 mm) at postretention (T3) (Table 2).

The mild group presented smaller changes (− 2.73 ± 2.18) than the severe group (− 9.47 ± 2.49) for mandibular LII during treatment even though LII changes showed high relapse values in both groups (mild group 3.08 ± 2.35 and severe group 4.12 ± 2.75). The severe group presented smaller changes for mandibular interpremolar width (− 1.66 ± 1.92 mm) than the mild group (− 4.04 ± 3.27) during the treatment period (Table 3).

**Table 3:** Dental arches dimensions changes over the time in the treatment time (T2-T1) and long-term follow-up time (T3-T2) (independent *t* tests)

Variables (mm)	Group mild crowding (*N* = 16)	Group severe crowding (*N* = 25)	*P*	Group mild crowding (*N* = 16)	Group severe crowding (*N* = 25)	*P*
	Mean (SD)	Mean (SD)		Mean (SD)	Mean (SD)	
	Treatment changes T2-T1			Long-term follow-up changes T3-T2		
*Maxillary dental casts measurements*						
Little irregularity index	− 6.48 (2.97)	− 8.29 (4.01)	0.129	1.18 (2.18)	2.59 (2.35)	0.062
3–3 width	0.56 (2.00)	0.62 (2.18)	0.934	− 1.90 (3.73)	− 0.85 (1.53)	0.214
5–5 width	− 1.39 (1.61)	− 0.96 (2.24)	0.514	− 1.50 (1.71)	− 1.16 (1.71)	0.541
6–6 width	− 1.11 (2.02)	− 1.30 (2.05)	0.770	− 0.65 (1.80)	− 0.34 (1.40)	0.560
Arch length	− 5.18 (4.33)	− 6.00 (3.13)	0.484	− 2.60 (3.59)	− 0.90 (2.44)	0.078
Arch perimeter	− 8.75 (6.80)	− 9.21 (4.71)	0.801	− 3.05 (2.04)	− 2.49 (3.81)	0.596
*Mandibular dental casts measurements*						
Little irregularity index	− 2.73 (2.18)	− 9.47 (2.49)	0.000*	3.08 (2.35)	4.12 (2.75)	0.222
3–3 width	0.20 (1.79)	1.42 (2.25)	0.076	-2.51 (2.72)	− 1.79 (2.01)	0.339
5–5 width	− 4.04 (3.27)	− 1.66 (1.92)	0.006*	-0.91 (2.11)	− 1.46 (2.05)	0.420
6–6 width	− 3.27 (2.21)	− 2.55 (2.33)	0.337	1.00 (2.25)	0.22 (2.78)	0.355
Arch length	− 5.25 (2.21)	− 4.49 (2.23)	0.295	-2.29 (1.55)	− 1.30 (2.32)	0.141
Arch perimeter	− 9.45 (5.44)	− 10.16 (2.71)	0.583	-3.72 (2.53)	− 3.14 (2.66)	0.495

^*^Statistically significant at *p* < 0.05

The sex, type of malocclusion, age at long-term posttreatment follow-up, retention time and initial mandibular crowding did not significantly influence the mandibular anterior crowding relapse (Table 4).

**Table 4:** Results of multiple linear regression analysis including the relapse of mandibular Little irregularity index (Md Little T3-2) as dependent variable and sex, type of malocclusion, age at long-term posttreatment follow-up, retention time and initial mandibular Little irregularity index as independent variables

*N* = 41	b*	Std.Err of b*	b	Std.Err of b	t(35)	*p*-value
Intercept			− 0.794	5.029	− 0.158	0.875
Sex	0.116	0.160	0.623	0.862	0.723	0.475
Type of malocclusion	− 0.056	0.184	− 0.293	0.969	− 0.303	0.764
Age at T3	0.119	0.176	0.056	0.083	0.679	0.502
Retention time	− 0.072	0.191	− 0.171	0.455	− 0.376	0.709
Md Little T1	0.345	0.192	0.221	0.123	1.796	0.081

Regression summary for dependent variable: Md Little T3-2

*R* = 0,340, *R*^2^ = 0,116 Adjusted *R*^2^ = - F(5,35) = 0,916

*p* < 0,482 Std Error of estimate: 2,635

## Discussion

This sample comprised 41 subjects treated with four premolars extraction, of which 35 were treated with four first premolar extractions, and 6 were treated with maxillary first and mandibular second premolar extractions. In our study, the mean value of 6 mm for mandibular LII at pretreatment was chosen to allocate the subjects into groups. This criterion was used to fulfill the sample size calculation [25, 26]. The rationale behind our study was that the greater the crowding at pretreatment, the greater the changes over the years. We included only patients treated with extraction in the sample to avoid treatment-related factors that could lead to bias. The sample treatment was carried out in the 1970s and 1980s when the diagnosis was based on cephalometric and occlusal features. At that time, facial analysis was not used. Therefore, the treatment with 4-premolar extraction and anchorage with headgear protocol was very usual, even in cases with mild crowding.

In this study, digital models were used, and measurements were taken using the Orthoanalyzer software. With the advancement of digital orthodontics, the use of digital models and measurements in studies is becoming increasingly common [3, 16, 27]. This software allows the cast image to be enlarged; therefore, the marking of points, lines and planes are more accurate. Recent studies have shown that digital models are as reliable as traditional plaster models, with high accuracy, reliability and reproducibility [21, 27, 28].

The mild crowding group showed a longer treatment time than the severe group. It could be justified because the mild group presented more subjects with Class II malocclusion, and the severe group presented more Class I subjects. Using headgear or Class II elastics to correct the anteroposterior relationship could have increased treatment time in the mild group [29]. The follow-up time was similar between the groups (Table 1).

The literature supports our results that report arch widths and length decreases after retention, whereas mandibular incisor irregularity increases [6, 8, 17]. The changes during treatment were related to the canine and *en masse* retractions. No intervention was performed between the end of the treatment and the long-term follow-up.

The main difference between groups at pretreatment was the severity of mandibular anterior crowding. The mandibular Little irregularity index of the severe group was approximately 3 times higher than the mild group (Table 2). Therefore, this measure was used to divide the groups. In addition, another significant difference between the groups at the pretreatment was the inter-second premolar distance. The mild group presented an inter-second premolar distance significantly greater than the severe group (39.43 ± 3.17 and 37.17 ± 2.20, respectively) (Table 2). This can probably be explained by the position occupied by the mandibular canines in the arch. In arches with greater crowding, the mandibular canines tend to occupy a more buccal position out of dental alignment. The result is that the premolars occupy a more mesial position in the arch. The more mesial the premolar is, the smaller its distance to its contralateral tooth. However, these differences were not significant at the end of treatment and in the long term (Table 2). The arch length was the main difference between groups after 37 years of treatment. The severe group had a significantly greater arch length than the mild group at long-term observation (16.93 ± 1.98 and 15.58 ± 1.76, respectively) (Table 2). Despite this statistical difference, the mean difference between groups is approximately 1.5 mm, which can be considered clinically irrelevant. It is known that the arch length decreases with age, and some studies found similar results [3, 5, 30].

Both groups showed acceptable maxillary alignment in the long term. This result is in accordance with Freitas et al*.* [4], where maxillary irregularity was more stable than the mandibular in the long term. The severe group showed significantly greater changes in mandibular anterior crowding at the end of treatment than the mild group (Table 3). This was expected since the severe group presented greater crowding at pretreatment, requiring greater changes for its correction. However, when evaluated 37 years after treatment, there was no statistically significant difference in the mandibular anterior crowding changes. In the long term, the changes were similar, irrespective of the crowding at the pretreatment.

It was previously demonstrated that the maxillary anterior crowding relapsed in the short term and remained stable in the long-term postretention. Also, the mandibular anterior crowding significantly decreased with treatment, showed a significant relapse in the short term and continued to significantly increase in the long-term postretention stage [4]. In our study, the retainers were removed in the short-term follow-up and may have influenced the LII results. Our results agree with previous studies, which state that a short period of using mandibular retainers does not seem to prevent long-term relapse. A classical paper states the differences between rapid and slow relapse. Although both are related to occlusal changes after orthodontic treatment, rapid relapse occurs due to periodontal remodeling, and slow relapse responds to late changes occurring during the postretention period [31]. It can be speculated that the long-term changes could be caused either by a maturational process or treatment and can occur whether or not a person has been under orthodontic treatment.

Despite the heterogeneity of our sample, the anterior crowding relapse in the long term was not influenced by sex, type of malocclusion, age at posttreatment evaluation (T3), retention time and initial LII (Table 4). Our findings are in agreement with classic studies that state that crowding relapse is multifactorial and unpredictable [22, 31]. According to Little et al. [32] and others, there are no variables such as degree of initial crowding, age, sex, angle classification, arch length, duration of treatment, maxillary and mandibular incisor proclination, horizontal and vertical growth amounts, mandibular plane angle that can reliably and certainly predict long-term relapse of anterior crowding [6, 33].

Little et al*. *[6] claimed that there was a trend toward moderate crowding at least ten years out of retention in orthodontically treated patients with the extraction of four premolars. However, the authors also found that patients with severe pretreatment crowding improved in the long term. In contrast, patients with satisfactory alignment (irregularity index less than 3.5 mm) before treatment tended to worsen over the years [6]. Ten to 20-year posttreatment, these authors found that the mean 10-years postretention increase in mandibular irregularity was 3.59 mm, and the corresponding value for the next 10 years was 0.77 mm [32]. They concluded that in patients treated with 4 premolars extractions, the maximum relapse occurred during the first 10-years postretention, and at 20-years postretention, only 10% of the treated sample could have their mandibular anterior irregularity clinically satisfactory [6, 32]. The present study showed a worsening of mandibular LII in both groups. Also, none of the patients presented perfect mandibular alignment at postretention, *i.e.,* none of the subjects had a Little irregularity index value of 0. Even so, the mild group showed 56.25% (9 subjects), and the severe group presented 56% (14 subjects) with satisfactory mandibular anterior alignment or minimal irregularity (1–3 mm) at the postretention stage. Six subjects in each group presented moderate irregularity (4–6 mm) at postretention, representing 37.5% of the mild group and 24% of the severe group. The mild group presented 6.25% (1 subject), and the severe group presented 16% (4 subjects) with severe irregularity (7–9 mm). One patient in the severe group (representing 4% of the group) had very severe irregularity at postretention (> 10 mm), almost 35 years after the removal of retainers. These findings agree with the current literature, which shows that the increase in incisor irregularity is a continuous phenomenon throughout the individual's life, also occurring in subjects who have not undergone orthodontic treatment [5]. Furthermore, despite the long-term changes in the incisors irregularity presented at T3, the correction of mandibular crowding with the extraction of 4 premolars showed satisfactory results over the years, irrespective of the amount of initial crowding.

After so many years after treatment, the changes in the incisors' irregularity and the dental arch dimensions are similar to the physiologic changes that occur during aging [2, 3, 16, 34]. According to Thilander [31] the continuing changes that occur after treatment cannot be distinguished from normal aging processes that occur regardless of whether a person has been treated orthodontically or not. One can say that a limitation of our study was the lack of an untreated control group or one that did not have crowding at pretreatment. Freitas et al*.* [5] compared the occlusal changes in orthodontically treated subjects 40 years after treatment with untreated controls. They found that LII increased in both groups over the years, but the changes were greater in the treated group [5]. Thus, the long-term increase in the mandibular incisor irregularity could be attributed to relapse and maturational occlusal changes. It is still difficult to determine which changes are caused by relapse from physiological aging. Normal permanent dentition maturational changes are generally similar in origin but are smaller in extent than orthodontically treated cases [5, 16, 34].

### Study limitations

The main limitation of our study is the heterogeneity of the sample. Although the groups were statistically comparable at pretreatment, we evaluated different populations composed of men and women with various malocclusions (Class I and Class II malocclusion) at pretreatment. The treatment modalities were also different, with some patients using Class II intermaxillary elastics or extra-oral headgear. The use of headgear for molar distalization and Class II correction was prevalent at that time. The extraction protocol varied among patients. While 35 patients were treated with four first premolar extractions, 6 were treated with maxillary first and mandibular second premolar extractions. In addition, it was not possible to know whether our results would be different if the sample had used bonded retainers for life. When the patients of our sample were treated, long-term use of retainers was not usual. Therefore there were no records of a long-term retrospective study. We suggest future long-term studies with retainers in place to evaluate the stability of the occlusal characteristics.

### Clinical implications

The life expectancy of the population is increasing. Teenagers and adults are starting orthodontic treatments with new appliances and technologies. The challenge of the orthodontist is to conduct an orthodontic treatment with excellent results and proportionate oral health-related quality of life for the patient. The knowledge of long-term outcomes is evidence-based information for the prognosis of future treatments.

Follow the oral health of patients with regularity, instructing them appropriately regarding the importance of using the retainers, their maintenance, and oral hygiene are the clinician's responsibility [8, 35].

## Conclusions

- Arch dimensions stability was similar in cases with initial mild and severe anterior irregularity.
- The mild crowding group presented proportionally more relapse of mandibular incisor irregularity than the severe crowding group in the long term. Even so, the correction of mild and severe crowding with the extraction of 4 premolars showed satisfactory results in the long term, even with the presence of maturational changes and relapse.

## Data Availability

The datasets used and/or analyzed during the current study are available from the corresponding author on reasonable request.

## Abbreviation

LII: Little’s irregularity index
